# Brain injuries: health care capacity and policy in Georgia

**DOI:** 10.5249/jivr.v13i1.1541

**Published:** 2021-01

**Authors:** Eka Burkadze, Nino Chikhladze, George Lobzhanidze, Nino Chkhaberidze, Corinne Peek-Asa

**Affiliations:** ^*a*^ Department of Public Health, Faculty of Medicine, Ivane Javakhishvili Tbilisi State University, 1 Chavchavadze Ave, 0179, Tbilisi, Georgia.; ^*b*^ Department of Occupational and Environmental Health, University of Iowa College of Public Health, 145 N. Riverside Drive 100 CPHB Iowa City, IA 52242.

**Keywords:** Georgia, Health care capacity, Head trauma, TBI prevention, TBI treatment resources, Traumatic brain injury

## Abstract

**Background:**

Over 90% of morbidity and mortality associated with traumatic brain injury (TBI) occurs in low- and middle-income countries. Lack of reliable, high-quality data regarding TBI prevention and care hinders the ability to reduce TBI burden. We sought to identify current TBI data collection practices and capacity in Georgia, focusing on pre-hospital, hospital, and rehabilitation treatment.

**Methods:**

The eight level I and two level II Trauma Hospitals in Georgia with the highest number of TBI admissions in 2017 were selected for study. A semi-structured survey about various aspects of TBI care was designed and semi-structured interviews of healthcare providers treating TBI patients (e.g. neurologists, neurosurgeons) were conducted based on this survey.

**Results:**

Pre-hospital triage protocols were not routinely used to match patient treatment needs with hospital capacity. All hospitals provided specialist care for TBI 24 hours/day. MRI was available at only three (30%) centers, and in-hospital rehabilitation units were available in only one (10%). No center used a defined protocol for treating TBI patients and no national protocol exists.

**Conclusions:**

Even among the largest, most highly specialized hospitals in Georgia, TBI care varies in terms of diagnostic and treatment protocols. While TBI specialists are available, diagnostic equipment often is not. Gaps in pre-hospital coordination and access to rehabilitation services exist and provide areas of focus for future investment in reducing TBI burden.

## Introduction

Traumatic brain injury (TBI) is an important public health issue for countries with emerging economies such as Georgia because health burden can negatively impact economic growth. Economic growth can also contribute to rising TBI rates. For example, Georgia has a growing transportation infrastructure and a rising rate of road traffic crashes, which are one of the leading causes of TBI.^1^ According to 2017 data obtained from the National Center for Disease Control and Public Health, 6918 TBI-related hospitalizations occurred in Georgia; 3441 (49.7%) of these patients were admitted in the capital city of Tbilisi.

TBI is often called a “silent epidemic” because its consequences can be long-lasting and are often not immediately apparent.^2,3,4^ Mild TBI and the cumulative impact of repetitive TBI, such as from some contact sports, are difficult to diagnose, also contributing to the silent nature of the TBI burden.^4,5^ At all levels of severity, TBI can cause temporary or permanent disability that is difficult to quantify at the individual, familial, or societal level. ^6^ Despite the challenges of quantifying the incidence and burden of TBI, it is clear that this condition impacts productivity and self-reliance, places a heavy burden on caregivers, and has enormous social cost.^7^

Emerging economies such as Georgia provide opportunities to integrate TBI prevention and treatment improvements as the health economy grows. From 1921 to 1995, the Georgian healthcare system was based on the Soviet Semashko model, and Georgian health authorities had very little input or control.^8,9^ With increased sovereignty in 1995 a new social insurance system and increased local control of healthcare delivery was established with assistance from the World Bank, World Health Organization (WHO), United Nations Children’s Fund (UNICEF), and American International Health Alliance(AIHA). From 2007 to 2012 the Georgian health care system became more decentralized and privatized, and in 2013, the government introduced a Universal Healthcare Coverage System that covers the entire country.^10,8^

Reducing the long-term consequences of TBI requires timely and organized care. To formulate a societal-level strategy for effective prevention and treatment, it is essential to understand current systems and to build evidence-based strategies using clinical data. However, in many developing countries, the reduction of TBI burden has been hindered by a lack of coordinated care and data on effective TBI prevention and treatment options. To fill this gap, our objectives were to identify TBI resources and treatment practices in the main hospitals treating TBI, to describe data collection capacity, and to identify TBI leaders and stakeholders.

## Methods

The data reported here were collected as a part of the project INITIaTE: International Collaboration to Increase Traumatic Brain Injury Surveillance in Europe, funded by the United States National Institutes of Health and led by the University of Iowa and the Cluj School of Public Health (NIH/NINDS R21NS098850). The goals of the project are to identify current TBI prevention and treatment capacity and to establish TBI data registries in Armenia, Georgia, and Moldova. Here we report on the first goal for the country of Georgia.

This mixed-method project collected information using country-based healthcare data and key informant interviews to describe TBI national leadership and treatment capacity at the pre-hospital, acute care, and rehabilitation phases. County-based healthcare data was pulled in 2017 and key informant interviews were conducted in 2018.

**Hospital Selection**

Study hospitals were identified based on a national patient census and included hospitals with the highest number of TBI admissions in 2017. All trauma hospitals were located in the capital city of Tbilisi. In 2017, hospitalizations in Tbilisi numbered 3441, and 10 hospitals accounted for 80% (2785 patients) of admissions (Table 1). These hospitals were selected for participation. Hospitals were categorized into levels of trauma-care capacity according to the classification system of the American Trauma Society. Eight were categorized as level 1 trauma centers and two were classified as level 2.

**Table 1:** Distribution of TBI Hospitalization Cases, Tbilisi 2017.

Facility	Number of TBI hospitalizations	% of total TBI hospitalizations
Amtel Hospital	466	14
Gudushauri Clinic	362	11
High Technology Medical Center, University Clinic	360	10
Elizabeth Blackwell Hospital	349	10
Emergency, Surgery and Traumatology Center	265	8
1st Hospital	255	7
The First University Clinic	219	6
Lancet Medical Center	199	6
Archangel St. Michael Multi Profile Clinical Hospital	167	5
National Center of Surgery	143	4

Source: National Center for Disease Control and Public Health, Georgia

**Survey Development**

A semi-structured survey was designed to cover various aspects of TBI care with questions pulled from previous literature and from consultation with the project Board of Advisors, which included anesthesiologists, emergency department physicians, epidemiologists, neurologists, and neurosurgeons. Interview questions addressed pre-hospital care, in-hospital care, rehabilitation care, hospital and country-level guidelines and protocols for treatment, data collection practices, and the government agencies and professional organizations leading TBI prevention and care. The survey was translated into the Georgian language and terminology was reviewed by the field specialists, who provided feedback on the initial formulation of questions.

**Data Collection**

At each hospital, the key physician identified by the hospital administration as being the lead in TBI care was interviewed. Individuals included two neurosurgeons, one neurologist, three anesthesiologists, and four emergency physicians. Each of the ten respondents agreed to complete the survey with audiotaping, and gave verbal consent for the interview. Participants were read the survey questions and answers were recorded.

**Analysis**

Quantitative and open-ended responses to survey questions were collected through notes taken during the interview and transcriptions of audio recordings. Open-ended questions were coded to identify separate themes, and themes were reviewed to identify common responses. Coding was conducted by hand and validated through multiple entry.

## Results

**Pre-hospital Care**

Participants were asked to identify if the country or region has a pre-hospital trauma system that provides standardized triage and transport of patients based on their clinical needs. Participants were then asked to describe the process used for patient transport to emergency hospitals. All participants responded that Georgia has no pre-hospital trauma system. Pre-hospital triage and mobilization of emergency services are provided by an Emergency Response Center, which is accessed by the public using the country-wide number “112.” The Emergency Response Center perform initial triage and mobilize field care services. This model is similar to the U.S. model of emergency management and is in contrast to the European model in which hospitals provide response teams. 

Decisions about transport to trauma hospitals are not based on severity of injury or clinical need of the patient. Every hospital with emergency services, both private and state-owned, is obliged to have written agreements with emergency ambulance services. Ambulances transport patients based on which hospital they have agreements with and in coordination with the Emergency Response System which will identify the nearest hospital the ambulance service work with. 

TBI patients most commonly arrive at hospitals by ambulance. A few arrive by private vehicles and, in rare cases, by a helicopter operated by the Ministry of Internally Displaced Person from the occupied territories, Labor, Health and Social Affairs of Georgia (MOH). All participants reported that patient transfers seldom occur between trauma hospitals because they all provide trauma services; when they do occur it is because of insurance issues. However, it is common for patients to be transferred from rural areas and smaller towns to Tbilisi, in particular from basic-care medical facilities, to specialized-care facilities. 

When asked about protocols to ensure that patients receive appropriate triage, field care, and transport, all respondents stated that there are no standardized protocols. Hospital personnel, including specialists such as neurosurgeons, reported that they are not consulted about which hospital patients are transported to.

**Acute Care**

Responses regarding existing frameworks for treatment and care of TBI patients were varied, with 50% of interviewees responding that existing frameworks for treatment and care are based on international requirements and guidelines; 30% that existing frameworks are based on national requirements and that patients are generally assessed and treated by neurosurgeons; and 20% stated that no frameworks exist and hospitals use their own guidelines. All participants indicated that the majority of TBI patients are admitted to hospital through the emergency department.

All of the participating hospitals provide care for TBI patients 24 hours a day and seven days a week. Among the eight adult hospitals, six (75%) had neurologists and 7 (87.5%) had neurosurgeons in-house at all times. Of the two children’s hospitals, both had neurologists in-house at all times and one had neurosurgeons in-house at all times. Radiologists were available in-house for all hospitals, while ear/nose/throat specialists were on-call for seven (87,5%) of adult and one (50%) of children’s hospitals (Table 2). Computed Tomography Scanning (CT) was available in all 10 hospitals, whereas Magnetic Resonance Imaging (MRI) was available only in three of them. 

**Rehabilitation**

Rehabilitation services represent an important gap in TBI care in Georgia. The majority of respondents (90%) indicated that rehabilitation services were available only in some clinics and not in the interviewees’ hospitals. The one respondent (10%) whose clinic had rehabilitation services noted that the services are limited to physiotherapy with little opportunity for cognitive therapy. Two participants (20%) noted that neither the government or private insurance finances rehabilitation services, and two participants (20%) also stated that the quality and financial availability of rehabilitation services are serious problems. All participants mentioned that access to rehabilitation services was limited for all people, but that TBI patients who live outside of the capitol city have almost no access to services.

**Gaps in TBI Prevention and Care**

The most pressing gaps identified for TBI care were related to pre-hospital care, rehabilitation care, and infrastructure for providing in-hospital care. Access to specialists and quality of care by specialists were not identified as a gap. Reasons for deficiencies in care for TBI patients included: a lack of access to current evidence through access to medical journals or international decision-making committees; a lack of medical equipment for diagnosis (such as CT scan availability); a lack of financial resources; and lack of rehabilitation services. 

Participants were also asked to identify gaps in prevention. Participants identified lack of priority of safety in relation to road traffic and workplace safety; lack of country-level policies requiring use of safety equipment such as helmets; and lack of a safety culture to promote safe behaviors.

**National Leadership for TBI Treatment and Prevention**

Responses regarding which national agency is responsible for leadership in TBI treatment were inconsistent. The Ministry of Internally Displaced Person from the Occupied territories, Labour, Health and Social Affairs of Georgia (MOH) was identified as the lead country agency by six (60%) of respondents, the Georgia Neurosurgery Society by two (20%), and the Shota Rustaveli National Science Foundation by 2 (20%). The Georgia Neurosurgical Society was identified by most respondents as the lead professional organization for physicians who treat TBI, but only 20% of respondents reported being members. All interviewees stated that no existing policies or legal frameworks at the national level regulate the treatment and care of TBI patients, and that none of the agencies identified as the country lead in TBI convened physicians to discuss treatment protocols or advocated for prevention strategies.

**Table 2:** Hospital Services for Traumatic Brain Injury Care, Georgia.

Access to specialized services	Adult Hospital N(%)	Children’s Hospital N (%)
**Neurologists**		
24/7	6(75%)	2(100%)
	On call	2(25%)
**Neurosurgeons**		
24/7	7(87.5%)	1(50%)
On call	1(12.5%)	1(50%)
**Radiologists **		
24/7	8(100%)	2(100%)
On call		
**Ear/Nose/Throat (ENT)**		
24/7	1(12.5%)	1(50%)
on call	7(87.5%)	1(50%)

## Discussion

In Georgia, total health care expenditures are increasing every year, demonstrating rising demand for health services. The share of total health expenditures in GDP (%) is reasonably high; Georgia spends on healthcare practically as much as the European Region’s high income countries (8%-9%).^8^ Although the government covers much of the care, there are still very high out of pocket payments which results in a heavy burden for households. Since 2006 the number of physicians per 100,000 population has been growing at a higher rate than in the European region.^8,9^ In contrast, from 1998 to 2013 the number of nurses per 100,000 population decreased and is lower than in European region and the CIS countries.^9,11^ Georgia is ranked second to last among European countries in the ratio of the number of nurses to physicians. Given the growth in the medical sector, opportunities exist to strengthen capacity for TBI treatment and care.^11^

The participants in this survey, who represent the ten leading hospitals providing TBI care in Georgia, identified some common strengths and gaps in TBI care and capacity. These hospitals are high-volume, level I and II trauma centers located in urban areas. Nationally, Georgia has no defined framework for triage and treatment of TBI patients. The fact that most respondents were not aware of the designated national lead agency on TBI treatment and care suggests that a lack of organization and cohesion of services could contribute to many of the observed gaps in care. 

Hospital staff generally reported having full access to specialists and some diagnostic equipment needed to treat TBI patients, but it was apparent that the approach to care differs across hospitals. Respondents reported following different guidelines, variably noting local, national, or international guidelines. They reported no policies or legal frameworks to regulate treatment of TBI patients at a national level. Hospitals varied slightly with respect to their emergency-department organization, hospital facilities, treatment practices, leadership, and existing policies on TBI. 

The ineffectiveness of rehabilitation services was the largest gap identified in TBI care. Others included access to services, quality of care, and financing of care. Also, given that all of the services were within one the large hospitals of a single city, patients living in rural areas are likely to have no access to TBI rehabilitation services. 

Our study has limitations and strengths. Our survey took approximately 45 minutes, which is a significant time commitment for a specialist physician. Long questionnaires have been associated with lower data quality, mainly due to boredom of the respondents. A second potential limitation is that our results are likely not generalizable to other hospitals in Georgia, given that the hospitals were selected because they treated the largest TBI volume, and are not likely to be generalizable to other countries that have their own systems of care. We had one key informant in each hospital and responses may have differed with other participants. Study strengths included the engaged process of developing the survey actively involving international partners and experts in the field. The survey was the first of its kind to document TBI capacity in Georgia and included a wide spectrum of care.

## Conclusion

Even among the largest and most highly specialized hospitals in Georgia, variation in the structure and process of TBI care was reported. Particular gaps in pre-hospital triage and transport and in rehabilitation care were identified. Given growth in the health sector in Georgia, opportunities to fill gaps and standardize care are present.
